# The Power of an Infant's Smile: Maternal Physiological Responses to Infant Emotional Expressions

**DOI:** 10.1371/journal.pone.0129672

**Published:** 2015-06-11

**Authors:** Sanae Mizugaki, Yukio Maehara, Kazuo Okanoya, Masako Myowa-Yamakoshi

**Affiliations:** 1 Graduate School of Education, Kyoto University, Kyoto, Japan; 2 Faculty of Education, Nagasaki University, Nagasaki, Japan; 3 Department of Cognitive and Behavioural Sciences, The University of Tokyo, Tokyo, Japan; University of Rennes-1, FRANCE

## Abstract

Infant emotional expressions, such as distress cries, evoke maternal physiological reactions. Most of which involve accelerated sympathetic nervous activity. Comparatively little is known about effects of positive infant expressions, such as happy smiles, on maternal physiological responses. This study investigated how physiological and psychological maternal states change in response to infants’ emotional expressions. Thirty first-time mothers viewed films of their own 6- to 7-month-old infants’ affective behavior. Each observed a video of a distress cry followed by a video showing one of two expressions (randomly assigned): a happy smiling face (smile condition) or a calm neutral face (neutral condition). Both before and after the session, participants completed a self-report inventory assessing their emotional states. The results of the self-report inventory revealed no effects of exposure to the infant videos. However, the mothers in the smile condition, but not in the neutral condition, showed deceleration of skin conductance. These findings demonstrate that the mothers who observed their infants smiling showed *decreased* sympathetic activity. We propose that an infant’s positive emotional expression may affect the branch of the maternal stress-response system that modulates the homeostatic balance of the sympathetic and parasympathetic nervous systems.

## Introduction

From the very first stage of the postpartum period, human caregivers and infants show unique forms of emotional interaction [1]. Infants show various emotional expressions such as crying and smiling, and their caregivers typically respond sensitively and automatically to these expressions. Many studies using various methodologies have investigated maternal physical and mental responses toward infants’ emotional expressions. For example, distress cries of infants evoke physiological responses in mothers, most of which involve accelerated cardiac activity, increased skin conductance, and a higher rate of respiration [2–6]. Several researchers have suggested that this physiological arousal caused by infant cries functions as ‘preparation for action’ [7–8]. When a mother finds that her infant is crying, she will approach, pick up, and attempt to console her infant. Mothers are quite frequently exposed to such physiologically stressful events during everyday parenting.

Given that these stressful responses are a fairly typical feature of parenting, how are such responses modulated, and can they be decreased? Levenson [9] proposed an interesting answer to this question. He suggested that positive emotions facilitate the process of recovery from physiological arousal provoked by negative emotions. This is called the *undoing* effect. Indeed, Fredrickson and Levenson [10] showed that cardiovascular activity induced by watching a negative film returns to baseline more quickly after watching a cheerful film than after a sad or neutral film. The undoing effect has been observed for visual images that elicit contentment and amusement following a fear-eliciting film [11], for pleasant pictures following unpleasant pictures [12], for pleasant music following disgusting pictures [13], and during a dyadic marital interaction with positive emotional behaviors [14].

The stimulation of positive emotions associated with the undoing effect may result in the restoration of homeostatic balance. Homeostasis is dependent on the dual operation of both sympathetic and parasympathetic autonomic nervous systems. When a person faces a stressful situation, sympathetic activity becomes dominant, causing an increase in skin conductance and heart rate, which helps prepare the person for an emergency. After the person is released from the stressful situation, parasympathetic activity becomes dominant and sympathetic activity decreases, with an associated reduction in skin conductance and heart rate, which is commonly associated with a person experiencing a (relatively) quiet, relaxed state. These homeostatic functions maintain the stability of the body’s internal environment in response to changes in the external environment.

The undoing effect and the restoration of homeostasis may occur in the course of daily childrearing experiences. Mothers experience stress reactions to their infants’ expressions of negative emotion, and subsequent positive emotional expressions of the infants may moderate or ameliorate these stress reactions. To our knowledge, however, few studies have investigated the undoing effect in early mother-infant interactions. Infant smiling is thought to be an important emotional expression that evokes maternal proximity and interactive social behavior [15–20]. Furthermore, caregiver responses to infant positive emotional expressions such as smiling and laughter play an important role in children’s cognitive and emotional development [21–23]. Although a number of researchers have suggested that positive infant emotional expressions are important for effective mother-infant interaction, exactly how infant smiling affects maternal physiological states remains unknown.

Some functional magnetic resonance imaging (fMRI) studies have reported that infants’ positive emotional expressions activate various maternal brain regions, including those underlying the reward system, motor planning, and inhibition of negative emotion; the activation of these regions is thought to be necessary to initiate positive parenting behaviors [24–26]. However, these studies emphasized the effects of infant smiling on changes from a calm maternal state. It is possible infant smiling might have stronger recovery effects on physiologically negative stressful states, by means of the undoing effect. These studies could therefore have underestimated the positive effects of infant smiling on maternal physiological states. Thus, it is necessary to examine whether and how infant smiling brings about positive recovery effects on mothers' physiologically stressful states, including those states caused by exposure to infants’ distress cries. In the present study, we investigated whether the happy smiling of infants attenuate their mothers' physiological responses to their preceding cries.

The present study focused on the effects of each mother’s individual daily parenting experiences with their own child? Some studies have suggested that maternal physiological reactivity to infant crying is associated with type of parenting behaviors [3,8,27]. Most studies have measured maternal physiological responses to infant emotional expressions by using standardized infant image stimuli, and not the images of the *mothers' own* infants. It is important to consider mothers’ physiological responses in response to their own infants’ emotional expressions, given that mothers’ physiological responses to the emotional expressions of their own infants are likely more ecologically valid in terms of predicting daily parenting behaviors.

In this study, mothers viewed videos of their own infants showing emotional facial expressions. Mothers were assigned randomly to one of two conditions. In both conditions, each mother observed a video showing her own infant’s distress cry; in the smile condition, this was followed by a video of her infant showing a happy smile and in the neutral condition, the infant in the video showing a calm, neutral expression. We predicted that the mothers’ sympathetic nervous system would be deactivated and/or their parasympathetic nervous system activated more rapidly when they saw their infant's happy smile following a distress cry rather than when they saw their infant with a neutral expression. Thus the mothers in the smile condition should show decreased heart rate and skin conductance relative to mothers in the neutral condition. In other words, the undoing effect should be observed when mothers experience their own infant smiling after they have experienced their own infant's distress cry.

## Method

### Participants

Thirty healthy right-handed mothers (mean age = 31.7 years, *SD* = 4.0, range = 24–43) and their first-born infants (14 girls and 16 boys, *M* age = 6.9 months, *SD* = 0.6, range = 5.6–7.9) participated in this study. The gestational age of each infant was greater than 36 weeks (*M* age = 39.2 weeks, *SD* = 1.2) and birth weight was greater than 2379 g (*M* weight = 3011 g, *SD* = 339). Data from four additional mothers were excluded from the analyses because of experimenter error during operation of the apparatus. All mothers and infants were free from psychiatric or neurological disorders and were not taking any medication.

The participants were recruited by telephone using Kyoto University’s computerized subject list. This study was approved by the Ethics Committee of Kyoto University (No. 25-P-17) and was conducted in accordance with standards specified in the 1964 Declaration of Helsinki. All mothers provided their written informed consent prior to participation (as specified in the *PLOS ONE* consent form).

### General procedure

Participants visited a laboratory at Kyoto University twice. During the first session, short scenes of each infant smiling and crying were videotaped after the mothers were told the purpose of this study. Approximately one week later, during the second session, the mothers took part in an experiment to measure their physiological changes while they watched video clips of their own infant. Physiological changes were indexed using heart rate, blood volume pulse, skin conductance, and respiration rate.

### Stimuli

During the first visit to the laboratory, each infant was videotaped in neutral, play, and separation situations. In the neutral situation, mothers were asked to keep their face neutral and direct their gaze away from their infant. In this situation, infants usually showed a neutral expression. In the play situation, mothers played peek-a-boo with their infants. In this situation, infants usually smiled and/or showed other pleased expressions. In the separation situation, mothers left their infant alone, moved to a space behind a curtain out of the infant’s sight, and kept silent. In this situation, infants were usually distressed and cried aloud. Participants completed either the neutral or the play situation prior to the separation situation. All infants wore a white dress that appeared the same as the others and sat in the same infant seat in front of a green partition board. The video camera was positioned about 1 m in front of the infant seat. From each videotaped play situation, we extracted a 30-sec portion of sustained footage during which each infant seemed most happy (‘smiling’ clip; S1 Movie). Similarly, 30-sec clips of each infant’s neutral expression (‘neutral’ clip; S2 Movie) and crying (‘crying’ clip; S3 Movie) were respectively obtained from the videotaped neutral and separation situations. We employed PMB software version 5.8 to edit these video clips (SONY Corporation, 2011). Based on facial affect coding methods described in previous studies [24, 28], two trained coders that were unaware of the purpose of the current study classified 60 video clips (30 crying, 15 neutral, and 15 smiling) into one of five affect groups: very happy, happy, neutral, sad, or very sad. High intercoder reliability was demonstrated in this classification (Pearson correlation coefficient: .967; *p* < .001).

**Fig 1:**
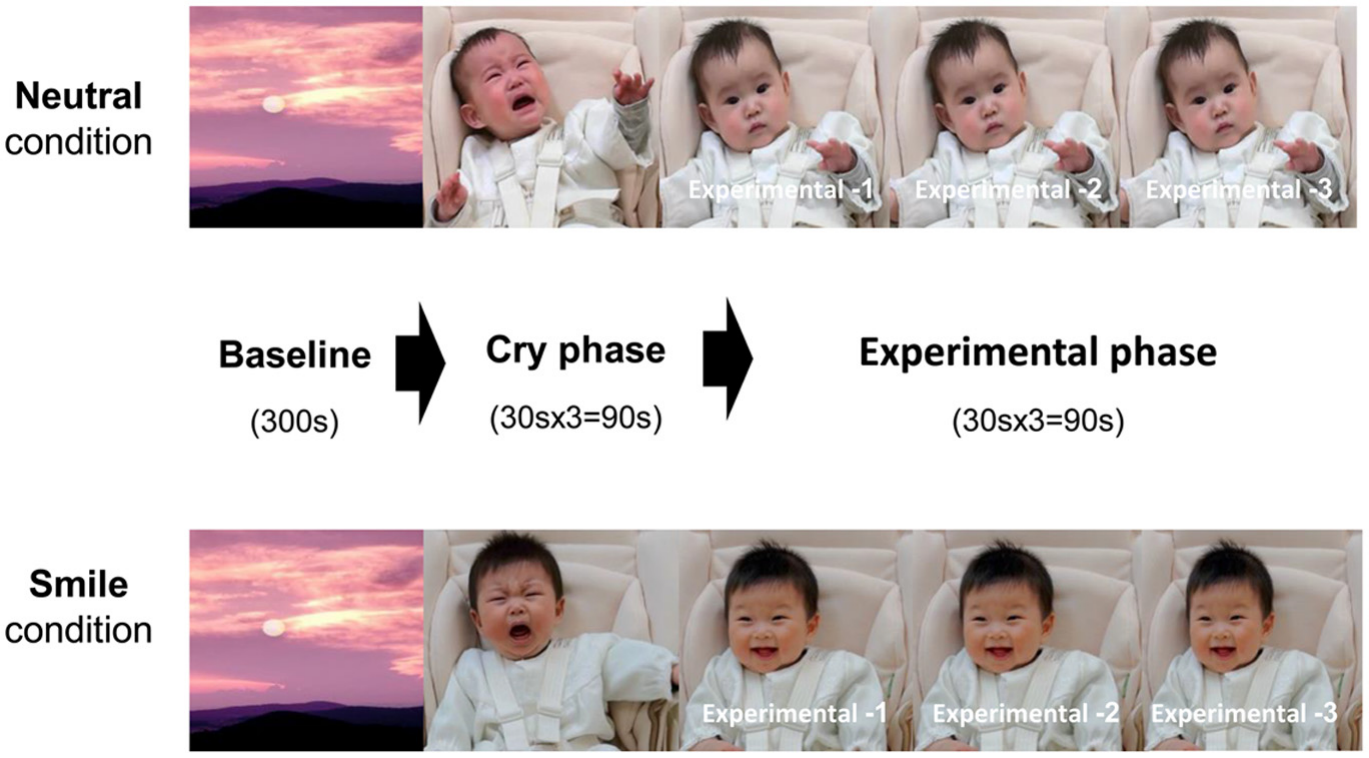
Schematic illustration of experimental procedure for each condition.

Between the participants’ first and second sessions we created audio-visual (A-V) test stimuli using the crying, smiling, and neutral clips, for use during the main experiment. An A-V stimulus consisted of the following three successive phases, as shown in Fig 1: (1) *a baseline phase* in which a relaxing video image of a beautiful mountain landscape was presented for 300 sec, (2) *a cry phase* during which the infant’s crying clip was played three times, for 90 sec in total, and (3) *an experimental phase* during which either the infant’s smiling clip or the neutral clip was presented three times, for 90 sec in total.

### Experimental procedure

Participants were given a brief introduction to the experimental procedure and were then seated in a comfortable chair approximately 60 cm from a 19-inch desktop monitor. Physiological states were recorded with the NeXus-10 Physiological Recording System (BioTrace+, Mind Media, the Netherlands), as described in the following section. Following preparation of the apparatus, the mother was asked to keep calm and watch the video stimuli presented on the monitor. The video stimuli were presented at the center of the monitor (1280 × 1024 pixels, 32 bit color) with volume control. The visual angle was about 24.5 × 22.6 degrees. As described above, mothers were randomly assigned to either the smile or neutral conditions as a between-participants factor, and these conditions were identical except for the final 90 sec of the video stimulus.

### Physiological measures

The NeXus-10 Blood Volume Pulse Sensor works with near-infrared light and measures the absorption of this light by the blood flowing through vessels, using the so-called photoplethysmography method. The sensor, which was attached to the left index finger, detects the peak blood volume pulse every time the heart beats. From the raw blood volume pulse signal, the *blood volume pulse amplitude* (BVPa) was calculated at a rate of 32 samples per second. Relative blood flow BVPa is affected by dilation and contraction of the blood vessels innervated by the sympathetic autonomic nervous system. *Heart rate* (HR) was calculated from the distance between the peaks of the blood volume pulse signal recorded at a rate of 32 samples per second. *Skin conductance response* (SC) was recorded at a rate of 32 samples per second with a NeXus-10 SC Sensor (bipolar Ag/AgCl dry electrodes). One SC finger electrode was attached to the middle finger and the other to the ring finger of the left hand. *Respiration rate* (RSP) per minute was calculated at a rate of 32 samples per second with a NeXus-10 RSP Sensor, which was placed on the participant’s abdomen by means of an elastic Velcro belt.

### Psychological measure

Previous studies have used the *Positive and Negative Affect Schedule* (PANAS) as a measure of the subjective emotional experience of a caregiver in response to infant behaviors [3,25]. The PANAS requires the respondents to indicate the extent to which they are feeling positive and negative emotions as expressed by various adjectives (e.g., *excited*, *interested*, *irritable*, and *nervous*). We employed the Japanese version of the PANAS [29], translated from the English version containing eight positive adjectives and eight negative adjectives, each rated on a 5-point scale [30]. Mothers rated their emotional states both before and after watching the video stimulus of their infant.

### Data analysis

All analyses were carried out using IBM SPSS Statistics 19. Quantile-quantile plots of residuals against fitted values were constructed to inspect for normality of all variables. To normalize the distribution of the values, a logarithmic transformation was performed on the skin conductance data [log (SC+1)]. For the *baseline phase*, data from the 5 sec interval immediately before the onset of the cry clip were averaged, and these averaged values were employed as baseline scores. For the *cry phase*, data were averaged over the 90 sec interval during which the cry clip was presented, and these averaged values were employed in further analyses. In order to examine whether infants’ cries brought about physiological changes in the mothers, we performed a two-way mixed analysis of variance (ANOVA) for each physiological measure, with a between-participants factor of condition (smile, neutral) and a within-participants factor of phase (baseline, cry). To discover the effects of infant expression following the cry phase, we examined detailed changes in maternal physiological status during the *experimental phase*. We divided the 90 sec experimental phases of infant smiling and neutral face presentations into three sub-phases of 30 sec each (experimental-1, -2, and -3) and calculated mean values for each sub-phase. In addition, we subtracted baseline scores from averaged values for the cry and experimental sub-phases to create change scores. For each physiological measure, a two-way mixed ANOVA with a between-participants factor of condition (smile, neutral) and a within-participants factor of phase (cry, experimental-1, -2, and -3) was conducted on the change scores.

For the psychological measures involving the PANAS, mean scores for the eight positive and eight negative items were calculated. Two-way mixed ANOVAs were conducted for the positive and negative scores separately, with a between-participants factor of condition (smile, neutral) and a within-participants factor of response point (pre-cry phase, post-experimental phase).

Effect sizes were reported as partial *η* ^2^ (eta squared), calculated to better interpret statistical findings. The Greenhouse-Geisser adjustment was applied if necessary to correct for sphericity, and epsilon along with corrected *p* values are reported, along with uncorrected degrees of freedom. In cases where a significant interaction effect or main effect was found by ANOVA, multiple Bonferroni comparisons were conducted.

## Results

### Effect of infant cry stimulation

The SC data of two mothers were excluded from the following analyses because of technical error, and another two were excluded because of excessive artifacts. BVPa data with excessive artifacts were also excluded for four mothers. Table 1 shows the means and standard deviations for the physiological measures at baseline and during the cry phase (for more detail see S1 Table). Two-way mixed ANOVAs revealed significant main effects of phase for BVPa (*F* (1, 24) = 14.50, *p* < .01, partial *η* ^2^ = .35), HR (*F* (1, 28) = 5.88, *p* = .02, partial *η* ^2^ = .17), RSP (*F* (1, 28) = 10.52, *p* < .01, partial *η* ^2^ = .27), and SC (*F* (1, 24) = 25.88, *p* < .001, partial *η* ^2^ = .49). Infant crying appears to have had a substantial influence on maternal physiological state. There was no significant interaction between the two conditions for any of the physiological measures used (BVPa: *F* (1, 24) = 2.74, *p* = .11, partial *η* ^2^ = .07; HR: *F* (1, 28) = 0.62, *p* = .44, partial *η* ^2^ = .02; RSP: *F* (1, 28) = 0.71, *p* = .41, partial *η* ^2^ = .02; SC: *F* (1, 24) = 3.13, *p* = .09, partial *η* ^2^ = .06) (for more detail see S2 Table). This confirms that the physiological effects of infant crying did not differ for the mothers in the two conditions.

**Table 1:** Means and standard deviations of physiological measures during baseline and cry phases.

		Condition		
Measure	Phase	Neutral	Smile	All participants
BVP (μV)	Baseline	42.12 (14.72)	58.75 (18.51)	50.43 (18.45)
	Cry	37.89 (11.17)	48.01 (9.10)	42.95 (11.24)
HR (bpm)	Baseline	71.87 (6.92)	70.63 (7.52)	71.25 (7.13)
	Cry	70.17 (6.39)	69.76 (7.19)	69.97 (6.68)
RSP (B/Min.)	Baseline	17.22 (3.70)	16.30 (3.51)	16.76 (3.57)
	Cry	18.79 (3.21)	18.98 (2.55)	18.88 (2.85)
SC (log(μS+1))	Baseline	0.38 (0.17)	0.33 (0.20)	0.36 (0.18)
	Cry	0.45 (0.20)	0.47 (0.26)	0.46 (0.22)

BVPa: Blood volume pulse amplitude (μV), HR: Heart rate (bpm: beats per minute), SC: Skin conductance response (log (μS+1)), RSP: Respiration rate (B/Min: breaths per minute). Standard deviations in parentheses.

### Physiological changes induced by infant smiling

**Fig 2:**
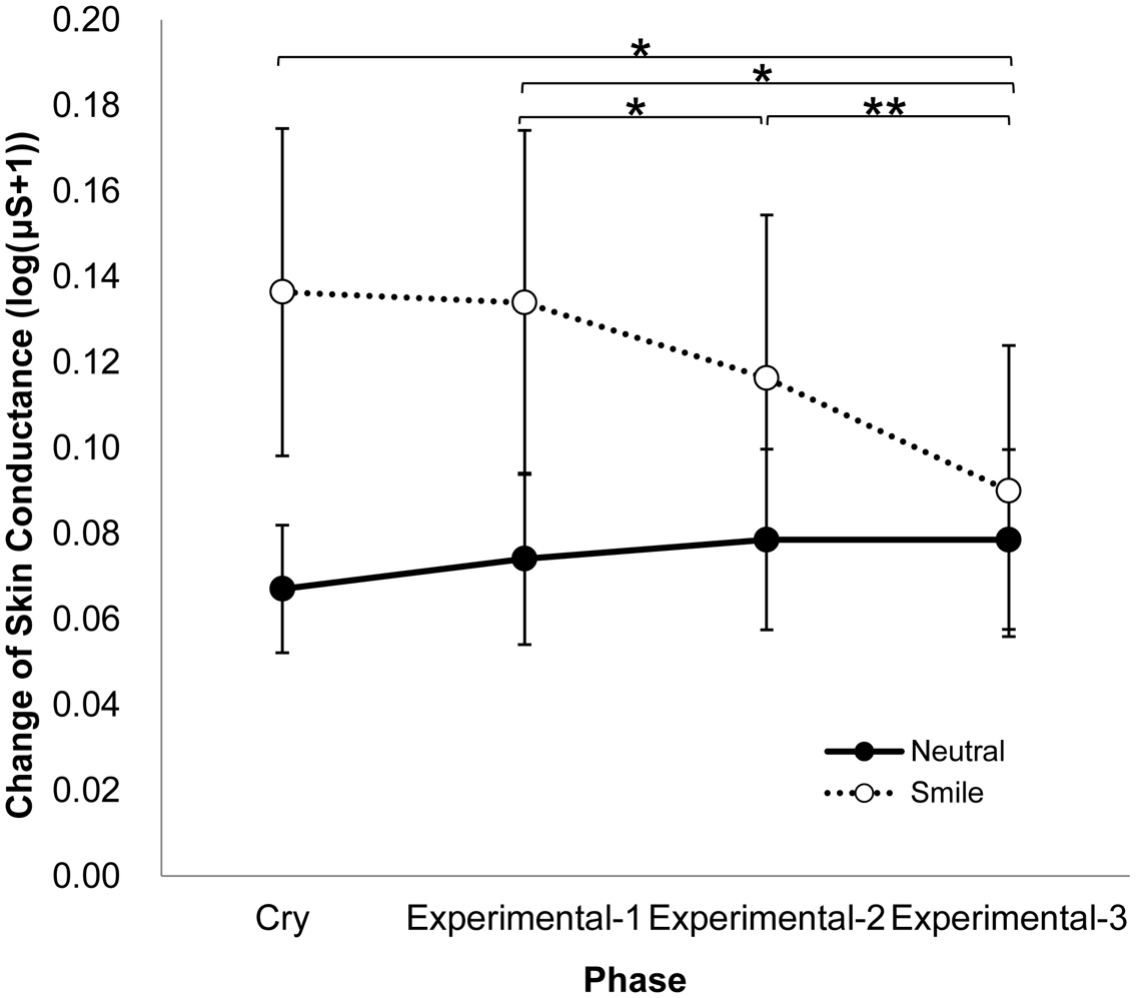
Means and SEMs for skin conductance change from baseline for each condition and phase. Means with an asterisk are significantly different, *p* < .05. Means with two asterisks are significantly different, *p* < .001.

Changes in physiological responses during the cry phase and the experimental phases are shown in Table 2 (for more detail see S3 Table). Skin conductance was relatively steady in the neutral condition but decreased in the smile condition. Two-way mixed ANOVAs (2 conditions: neutral, smile × 4 phases: cry, experimental-1, -2, and -3) revealed significant main effects of phase for BVPa and HR (*F* (3, 72) = 21.35, *p* < .001, partial *η* ^2^ = .46, and *F* (3, 84) = 5.49, *p* < .01, partial *η* ^2^ = .16, *ε* = .67, respectively). Physiological states of mothers substantially changed during the experimental phase, although no significant interaction was found for BVPa (*F* (3, 72) = 0.76, *p* = .52, partial *η* ^2^ = .02) or for HR (*F* (3, 84) = 0.22, *p* = .80, partial *η* ^2^ = .01, *ε* = .67). For RSP, there was no significant main effect of phase (*F* (3, 84) = 0.30, *p* = .82, partial *η* ^2^ = .01) and no significant interaction (*F* (3, 84) = 0.16, *p* = .93, partial *η* ^2^ = .01). Importantly, for SC, a significant interaction between condition and phase was observed (*F* (3, 72) = 22.42, *p* < .001, partial *η* ^2^ = .16, *ε* = .53) (for more detail see S4 Table). Fig 2 shows the SC changes for each condition and phase. Although there this figure appears to show be a meaningful difference between the smile and neutral condition in the cry phase in Fig 2, the difference did not reach significance (Bonferroni correction, *p* = .095). Further subsidiary analyses revealed that SC of mothers in the smile condition significantly decreased from the experimental-1 phase to the experimental-2 phase (Bonferroni correction, *p* < .05) and further to the experimental-3 phase (Bonferroni correction, *p* < .001). Mothers in the smile condition also showed a significantly lower SC during the experimental-3 phase than during the cry phase (Bonferroni correction, *p* < .05), while such a difference was not observed in the neutral condition.

**Table 2:** Means and standard deviations of physiological measures (change scores) during cry and experimental phases.

		Phase			
Measure	Condition	Cry	Experimenal-1	Experimenal-2	Experimenal-3
BVPa	Neutral (n = 13)	93.90 (20.32)	105.37 (23.82)	108.27 (21.55)	108.82 (19.69)
	Smile (n = 13)	84.80 (13.30)	100.03 (17.22)	100.58 (17.28)	97.13 (18.55)
HR	Neutral (n = 15)	-1.88 (2.39)	-3.47 (3.27)	-3.35 (3.11)	-2.77 (2.98)
	Smile (n = 15)	-1.05 (3.32)	-2.89 (3.90)	-2.09 (4.37)	-1.72 (4.47)
RSP	Neutral (n = 15)	1.78 (3.96)	1.47 (3.83)	1.39 (3.45)	1.64 (3.16)
	Smile (n = 15)	2.68 (3.31)	2.47 (4.67)	2.30 (5.24)	2.06 (4.90)
SC	Neutral (n = 13)	0.07 (0.05)	0.07 (0.07)	0.08 (0.08)	0.08 (0.08)
	Smile (n = 13)	0.14 (0.14)	0.13 (0.14)	0.12 (0.14)	0.09 (0.12)

Change scores (except BVPa) were calculated by subtracting baseline scores from averaged values for the cry and experimental sub-phases. Change scores for BVPa were calculated by dividing averaged values for the cry and experimental sub-phases by baseline scores. Standard deviations in parentheses.

### Subjective emotional experience

Table 3 shows mean PANAS scores for each film condition (smile, neutral) and each response point (pre-cry phase, post-experimental phase) (for more detail see S5 Table). A two-way mixed ANOVA on positive affect scores did not reveal significant main effects or a significant interaction. Another two-way mixed ANOVA on negative affect scores showed that scores at the point of the post-experimental phase were significantly higher than those at the point of the pre-cry phase (*F* (1, 28) = 12.81, *p* < .01, partial *η* ^2^ = .29), but the interaction did not reach significance (*F* (1, 28) = 3.16, *p* = .09, partial *η* ^2^ = .07). Further, there was no significant difference between the conditions for negative affect scores (*F* (1, 28) = 0.04, *p* = .84, partial *η* ^2^ < .01) (for more detail see S6 Table). Therefore, mothers' subjective feelings did not differ as a function of the video condition.

**Table 3:** Means and standard deviations of psychological measures at the response points of pre-cry and post-experimental phases.

		Condition		
Measure (PANAS)	Response Point	Neutral	Smile	All participants
Positive	Pre-Cry	2.19 (0.75)	2.49 (0.75)	2.34 (0.75)
	Post-Experimental	2.03 (0.87)	2.33 (0.81)	2.18 (0.84)
Negative	Pre-Cry	1.25 (0.43)	1.48 (0.56)	1.37 (0.51)
	Post-Experimental	2.09 (0.86)	1.77 (1.03)	1.93 (0.94)

Standard deviations in parentheses.

## Discussion

Parenting always involves exposure to a series of infant expressions, reflective of negative and positive emotions, which are accompanied by maternal physiological and psychological arousal. How are such maternal responses modulated? The aim of this study was to examine a possible undoing effect of infant smiling on physiological and psychological maternal arousal caused by infant distress cries. We predicted that when mothers saw the happy smiling of their infants after previously being exposed to infant distress, sympathetic nervous system activity would be suppressed more rapidly than when the mothers experienced neutral expressions. The results showed no clear effect on subjective self-reports of emotional feelings (PANAS) as a function of which video, smiling or neutral, was viewed. On one hand, we found remarkable changes in maternal physiological state during the experimental phase, in which they viewed infant smiling or neutral expressions. Infant smiling did indeed suppress sympathetic nervous activities after exposure to infant distress cries. On the other hand, physiological arousal induced by infant distress crying was sustained when mothers did not experience positive emotional expressions from their infants. This evidence suggests that infant smiling may help restore mothers’ homeostatic balance.

After exposure to their own infant's distress cry, mothers in the smile condition showed a significant deceleration of skin conductance as they observed their infants smiling, while mothers in the neutral condition showed no such change. This result is consistent with prior research showing that the undoing effect is caused by positive emotional stimuli [11, 13]. However, for subjective emotional experience, we did not find a significant difference in positive emotion between the neutral and smiling conditions. If this is the case, why does the smiling of infants cause a decrease in maternal skin conductance? In general, skin conductance is considered to represent levels of arousal in response to emotional situations [31, 32]. Strong emotional experience causes the release of acetylcholine within the sympathetic nervous system. This neurotransmitter release during a high arousal state is related to an increase in eccrine sweat gland activity, which causes acceleration of skin conductance. The acceleration of skin conductance is thought to represent a function of ‘preparation for action’ known as the fight or flight response (i.e., attack and escape behavior). In the present study, skin conductance was significantly reduced while viewing infant smiling compared to viewing neutral infant faces. It is possible that mothers felt more safe and relaxed while viewing infant smiling than while viewing neutral faces.

Our results may also be explained from the perspective of emotion regulation. As mentioned in the introduction, infant smiling is thought to evoke maternal proximity and interactive social behavior [15, 16]. Infant smiling might arouse in mothers complex cognitive processes that work to attenuate their skin conductance responses. Regarding this possibility, previous studies of emotion regulation have reported that demanding cognitive tasks attenuate preceding emotional responses. For example, Erber and Tesser [33] showed that participants who completed a mathematical task after observing a sad movie were in a less sad mood than participants in a group who did not complete the task. Furthermore, cognitive tasks attenuate not only the emotional mood but also the physiological responses [34, 35]. In daily parenting situations, mothers contingently deliver smiling, gazing, vocalizing, and touching to keep the infant’s attention and to encourage speech-like sounds [17, 19, 20]. In order to choose the most appropriate behavioral response to their infants, mothers need to integrate both affective and cognitive information about current infant states. An fMRI study reported by Strathearn and colleagues [24] showed that an extensive brain network is activated when first-time mothers see their own infant’s face. This network is associated with affective and cognitive processes directed toward motor/behavioral output. They also reported that maternal brain regions associated with the reward-processing system were significantly activated by happy (but not neutral or sad) faces of their own infant. It is possible that infant smiling may facilitate certain brain activities and, as a result, enhance several kinds of cognitive functioning, which leads to adequate parenting behavior. Further research is needed to determine whether such neurocognitive processes are related to the attenuation of maternal skin conductance responses.

In the present study, no significant interactions were found between condition and phase for BVPa, HR, or RSP. One possible explanation is that a neutral image of the infant is enough of a positive stimulus to evoke maternal positive emotion. Mothers in the neutral condition may have felt safe watching the video clip in which their own infant was sitting calmly, in much the same way as mothers in the smile condition would feel. Hence, in the current study, the difference between the smile and neutral conditions may be so subtle that is difficult to detect meaningful effects of infant smiling on BVPa, HR, and RSP. Further, infant emotional intensity in some of the crying and smiling clips was weaker than the others, and the extent to which infants expressed emotion in the video clips may have influenced the magnitude of maternal physiological responses. Future research should control emotional valence across infants by having mothers estimate the intensity of their infants’ emotions in order to clarify differential influences of smiles and neutral expressions on the mothers’ physiological state.

Although the present study recruited mothers with first-born infants, further studies will be needed to investigate whether maternal physiological responses to infant smiling varies with stage of motherhood or parental experience. Previous studies have implied that teen mothers are less likely than adult mothers to show physiologically heightened responses to infant cries [8]. Non-parents have showed more cardiac reactivity than parents to infant crying [5]. Studies that consider life stages and childrearing experience will therefore provide more insights into the physical and cognitive mechanisms underlying stressful states and associated recovery processes. Indeed, individual differences in maternal physiological reactivity to infant-related stimuli are associated with maternal sensitivity to infant emotional behavior [27, 36, 37]. Thus, it is likely that different parenting experiences may result in varying influences of infant smiling on maternal physiological states.

In summary, our results reveal that infant smiling plays an important role in moderating the physiological stress caused by infant crying that mothers experience in everyday parenting. The present study provides an understanding of the physiological basis of childrearing stress. If it is the case that watching infant smiling reduces maternal physiological stress, the current results can be applied in the search for better approaches to help parents manage daily childrearing stress. For instance, depictions of infants’ positive smiling may relieve the stress of daily childrearing. Further studies are necessary to develop such potential future applications that will decrease parental childrearing stress.

## Supporting Information

S1 Movie Movie sample of an infant smiling. (MP4)

S2 Movie Movie sample of an infant neutral face. (MP4)

S3 Movie Movie sample of an infant crying. (MP4)

S1 Table Descriptive statistics of physiological measures during baseline and cry phases for Table 1. (PDF)

S2 Table Results of two-way ANOVA for Table 1. (PDF)

S3 Table Descriptive statistics of physiological measures (change scores) during cry and experimental phases for Table 2. (PDF)

S4 Table Results of two-way ANOVA (related to Fig 2). (PDF)

S5 Table Descriptive statistics of psychological measures at response points (pre-cry and post-experimental phases). (PDF)

S6 Table Results of two-way ANOVA for Table 3. (PDF)

## References

[1] Myowa-Yamakoshi M (2010) Early social cognition in chimpanzees (Pan troglodytes) In: Suddendorf E, Ross S, Matsuzawa T (Eds.) The mind of the chimpanzees. Chicago: The University of Chicago Press pp. 23–31.

[2] Crowe HP, Zeskind PS (1992) Psychophysiological and perceptual responses to infant cries varying in pitch: Comparison of adults with low and high scores on the Child Abuse Potential Inventory. Child Abuse Negl, 16: 19–29. 1544026 10.1016/0145-2134(92)90005-c

[3] Vecchio TD, Walter A, O'Leary SG (2009) Affective and physiological factors predicting maternal response to infant crying. Infant Behav Dev 32: 117–122. 10.1016/j.infbeh.2008.10.005 19081636

[4] Groh AM, Roisman GI (2009) Adults' autonomic and subjective emotional responses to infant vocalizations: The role of secure base script knowledge. Dev Psychol 45: 889–893. 10.1037/a0014943 19413441

[5] Out D, Pieper S, Bakermans-Kranenburg MJ, van Ijzendoorn MH (2010) Physiological reactivity to infant crying: a behavioral genetic study. Genes Brain and Behav 9: 868–876. 10.1111/j.1601-183X.2010.00624.x 20618442

[6] Wolfe DA, Fairbank JA, Kelly JA, Bradlyn AS (1983) Child abusive parents' physiological responses to stressful and non-stressful behavior in children. Behav Assess 5: 363–371.

[7] Furedy JJ, Fleming AS, Ruble D, Scher H, Daly J, Day D, et al (1989) Sex-differences in small-magnitude heart-rate responses to sexual and infant-related stimuli―a psychophysiological approach. Physiol Behav 46: 903–905. 2629003 10.1016/0031-9384(89)90056-5

[8] Giardino J, Gonzalez A, Steiner M, Fleming AS (2008) Effects of motherhood on physiological and subjective responses to infant cries in teenage mothers: A comparison with non-mothers and adult mothers. Horm Behav 53: 149–158. 18076883 10.1016/j.yhbeh.2007.09.010

[9] Levenson RW (1988) Emotion and the autonomic nervous system: A prospectus for research on autonomic specificity, in Social psychophysiology and emotion: Theory and clinical applications. Oxford: John Wiley & Sons pp.17–42.

[10] Fredrickson BL, Levenson RW (1998) Positive emotions speed recovery from the cardiovascular sequelae of negative emotions. Cognd Emot 12: 191–220. 21852890 10.1080/026999398379718 PMC3156608

[11] Fredrickson BL, Mancuso RA, Branigan C, Tugade MM (2000) The undoing effect of positive emotions. Motiv Emot 24: 237–258. 21731120 10.1023/a:1010796329158 PMC3128334

[12] Fujimura T, Katahira K, Okanoya K (2013) Contextual modulation of physiological and psychological responses triggered by emotional stimuli. Front Psychol 4: 212 10.3389/fpsyg.2013.00212 23675359 PMC3650463

[13] Sokhadze EM (2007) Effects of music on the recovery of autonomic and electrocortical activity after stress induced by aversive visual stimuli. Appl Psychophysiol Biofeedback 32: 31–50. 17333313 10.1007/s10484-007-9033-y

[14] Yuan JW, McCarthy M, Holley SR, Levenson RW (2010) Physiological down-regulation and positive emotion in marital interaction. Emotion 10: 467–474. 10.1037/a0018699 20677864

[15] Bowlby J (1969) Attachment and loss: Vol.1 Attachment. London: Hogarth.

[16] Bowlby J (1982) Attachment and loss-Retrospect and prospect. Am J Orthopsychiatry 52: 664–678. 7148988 10.1111/j.1939-0025.1982.tb01456.x

[17] Koester LS, Papousek H, Papousek M (1989) Patterns of rhythmic stimulation by mothers with three-month-olds: A cross-modal comparison. Int Behav Dev 12: 143–154.

[18] Nwokah E, Fogel A (1993) Laughter in mother-infant emotional communication. Humor 6: 137–161.

[19] Nwokah EE, Hsu HC, Dobrowolska O, Fogel A (1994) The development of laughter in mother-infant communication: Timing parameters and temporal sequences. Infant Behav Dev 17: 23–35.

[20] Hsu HC, Fogel A, Messinger DS (2001) Infant non-distress vocalization during mother-infant face-to-face interaction: Factors associated with quantitative and qualitative differences. Infant Behav Dev 24: 107–128.

[21] Murray L (1992) The Impact of postnatal depression on infant development. J Child Psychol Psychiatry 33: 543–561. 1577898 10.1111/j.1469-7610.1992.tb00890.x

[22] Murray L, Cooper PJ (1997) EDITORIAL: Postpartum depression and child development. Psychol Med 27: 253–260. 9089818 10.1017/s0033291796004564

[23] Murray L, Cooper PJ, Hipwell A (2003) Mental health of parents caring for infants. Arch Women’s Ment Health 6: s71–s77. 14615925 10.1007/s00737-003-0007-7

[24] Strathearn L, Li J, Fonagy P, Montague PR (2008) What's in a smile? Maternal brain responses to infant facial cues. Pediatrics 122: 40–51. 10.1542/peds.2007-1566 18595985 PMC2597649

[25] Strathearn L, Fonagy P, Amico J, Montague PR (2009) Adult attachment predicts maternal brain and oxytocin response to infant cues. Neuropsychopharmacology 34: 2655–2666. 10.1038/npp.2009.103 19710635 PMC3041266

[26] Riem MME, van Ijzendoorn MH, Tops M, Boksem MAS, Rombouts SARB, Bakermans-Kranenburg MJ (2012) No laughing matter: Intranasal oxytocin administration changes functional brain connectivity during exposure to infant laughter. Neuropsychopharmacology 37: 1257–1266. 10.1038/npp.2011.313 22189289 PMC3306887

[27] Joosen KJ, Mesman J, Bakermans-Kranenburg MJ, Pieper S, Zeskind PS, van IJzendoorn MH (2013) Physiological reactivity to infant crying and observed maternal sensitivity. Infancy 18: 414–431.

[28] Cole PM, Barrett KC, Zahn-Waxler C (1992) Emotion displays in two-year-olds during mishaps. Child Dev 63: 314–324. 1611936

[29] Sato T, Yasuda A (2001) Development of the Japanese version of positive and negative affect achedule (PANAS) scales. JPN J Pers 9: 138–189.

[30] Watson D, Clark LA, Tellegen A (1988) Development and validation of brief measures of positive and negative affect: The PANAS scales. J Pers Soc Psychol 54: 1063–1070. 3397865 10.1037//0022-3514.54.6.1063

[31] Codispoti M, Bradley MM, Lang PJ (2001) Affective reactions to briefly presented pictures. Psychophysiology 38: 474–478. 11352135

[32] Critchley HD, Elliot R, Mathias CJ, Dolan RJ (2000) Neural activity relating to generation and representation of galvanic skin responses: a functional magnetic resonance imaging study. J Neurosci 20: 3033–3040 10751455 10.1523/JNEUROSCI.20-08-03033.2000 PMC6772223

[33] Erber R, Tesser A (1992) Task effort and the regulation of mood: The absorption hypothesis. J Exp Soc Psychol 28: 339–359.

[34] Iida S, Nakao T, Ohira H (2011) Implicit attenuation of subsequent emotion by cognitive activity. Cogn Affect Behav Neurosci 11: 476–484. 10.3758/s13415-011-0045-y 21617899

[35] Iida S, Nakao T, Ohira H (2012) Prior cognitive activity implicitly modulates subsequent emotional responses to subliminally presented emotional stimuli. Cogn Affect Behav Neurosci 12: 337–345. 10.3758/s13415-012-0084-z 22373927

[36] Frodi AM, Lamb ME (1980) Child abusers' responses to infant smiles and cries. Child Dev 51: 238–241. 7363736

[37] Donovan WL, Leavitt LA, Balling JD (1978) Maternal physiological response to infant signals. Psychophysiology 15: 68–74. 625524 10.1111/j.1469-8986.1978.tb01337.x

