# Supplementary material for: The Power of an Infant's Smile: Maternal Physiological Responses to Infant Emotional Expressions
Source: PLoS One. 2015 Jun 11;10(6):e0129672. doi: 10.1371/journal.pone.0129672 (PMC4465828; doi:10.1371/journal.pone.0129672)
Supplement: S1 Table — (PDF) [file pone.0129672.s004.pdf]

S1 Table. Descriptive statistics of physiological measures during baseline and cry phases for Table 1.

			Condition			
			Neutral		Smile	
	**Source**		Baseline	Cry	Baseline	Cry
BVPa (μV)	Mean		42.12	37.89	58.75	48.01
	Std. Error of Mean		4.08	3.10	5.13	2.52
	Median		45.30	36.41	52.07	46.25
	Std. Deviation		14.72	11.17	18.51	9.10
	Variance		216.66	124.74	342.66	82.88
	Skewness		-0.01	0.68	0.78	-0.38
	Std. Error of		0.62	0.62	0.62	0.62
	Kurtosis		-1.14	0.61	-0.76	0.13
	Std. Error of		1.19	1.19	1.19	1.19
	Range		43.54	39.91	55.44	31.94
	Percentile	25	26.96	31.14	45.98	43.73
		50	45.30	36.41	52.07	46.25
		75	53.43	44.74	78.17	54.76
HR (bpm)	Mean		71.87	70.17	70.63	69.76
	Std. Error of Mean		1.79	1.65	1.94	1.86
	Median		71.27	71.47	72.27	72.27
	Std. Deviation		6.92	6.39	7.52	7.19
	Variance		47.83	40.79	56.55	51.63
	Skewness		-0.04	-0.23	0.03	-0.18
	Std. Error of		0.58	0.58	0.58	0.58
	Kurtosis		-0.80	-0.71	-1.58	-1.07
	Std. Error of		1.12	1.12	1.12	1.12
	Range		22.85	21.77	22.92	23.59
	Percentile	25	65.42	65.30	64.15	63.42
		50	71.27	71.47	72.27	72.27
		75	77.25	75.51	77.60	75.06
RSP (B/Min.)	Mean		17.22	18.79	16.30	18.98
	Std. Error of Mean		0.95	0.83	0.91	0.66
	Median		17.32	19.71	16.25	19.33
	Std. Deviation		3.70	3.21	3.51	2.55
	Variance		13.68	10.31	12.33	6.50
	Skewness		0.62	-0.80	-0.75	-0.84
	Std. Error of		0.58	0.58	0.58	0.58
	Kurtosis		0.08	0.33	2.07	0.13
	Std. Error of		1.12	1.12	1.12	1.12
	Range		13.36	11.71	14.64	8.42
	Percentile	25	14.28	17.93	14.88	17.11
		50	17.32	19.71	16.25	19.33
		75	19.32	20.52	18.90	21.52
SC (log(μS+1))	Mean		0.38	0.45	0.33	0.47
	Std. Error of Mean		0.05	0.05	0.06	0.07
	Median		0.33	0.40	0.24	0.43
	Std. Deviation		0.17	0.20	0.20	0.26
	Variance		0.03	0.04	0.04	0.07
	Skewness		0.63	0.54	1.04	0.37
	Std. Error of		0.62	0.62	0.62	0.62
	Kurtosis		-0.82	-0.70	-0.16	-1.18
	Std. Error of		1.19	1.19	1.19	1.19
	Range		0.50	0.61	0.60	0.73
	Percentile	25	0.23	0.28	0.17	0.20
		50	0.33	0.40	0.24	0.43
		75	0.53	0.61	0.50	0.70
