# Supplementary material for: The Power of an Infant's Smile: Maternal Physiological Responses to Infant Emotional Expressions
Source: PLoS One. 2015 Jun 11;10(6):e0129672. doi: 10.1371/journal.pone.0129672 (PMC4465828; doi:10.1371/journal.pone.0129672)
Supplement: S2 Table — (PDF) [file pone.0129672.s005.pdf]

S2 Table. Results of two-way ANOVA for Table 1.

		Type III Sum of Squares	df	Mean Square	F	Sig.	Partial Eta Squared
BVPa (μV)	Intercept	113370.56	1	113370.56	340.19	0.00	0.92
	Condition	2324.60	1	2324.60	6.98	*0.01	0.02
	Phase	727.97	1	727.97	14.50	*0.00	0.35
	Phase*Condition	137.41	1	137.41	2.74	0.11	0.07
	Error	1205.19	24	50.22			0.58
HR(bpm)	Intercept	299123.32	1	299123.32	3175.57	0.00	0.99
	Condition	10.27	1	10.27	0.11	0.74	0.00
	Phase	24.73	1	24.73	5.88	*0.02	0.17
	Phase*Condition	2.60	1	2.60	0.62	0.44	0.02
	Error	117.77	28	4.21			
RSP (B/Min.)	Intercept	19054.00	1	19054.00	1273.85	0.00	0.98
	Condition	2.00	1	2.00	0.13	0.72	0.00
	Phase	67.90	1	67.90	10.52	***0.00	0.27
	Phase*Condition	4.57	1	4.57	0.71	0.41	0.02
	Error	180.68	28	6.45			
SC (μS)	Intercept	8.61	1	8.61	106.10	0.00	0.82
	Condition	0.00	1	0.00	0.04	0.84	0.00
	Phase	0.13	1	0.13	25.88	***0.00	0.49
	Phase*Condition	0.02	1	0.02	3.13	0.09	0.06
	Error	0.12	24	0.01			0.45

Phase= {Baseline, Cry}, Condition={Neutral, Smile}

Sig.: Significance Probability, df: Degree of Freedom, *p<0.05, ***p<0.001
