# Supplementary material for: The Power of an Infant's Smile: Maternal Physiological Responses to Infant Emotional Expressions
Source: PLoS One. 2015 Jun 11;10(6):e0129672. doi: 10.1371/journal.pone.0129672 (PMC4465828; doi:10.1371/journal.pone.0129672)
Supplement: S3 Table — (PDF) [file pone.0129672.s006.pdf]

S3 Table. Descriptive statistics of physiological measures (change scores) during cry and experimental phases for Table 2.

			Condition							
			Neutral				Smile			
	**Source**		Cry	Exp1	Exp2	Exp3	Cry	Exp1	Exp2	Exp3
BVPa (%)	Mean		93.90	105.37	108.27	108.82	84.80	100.03	100.58	97.13
	Std. Error of Mean		5.63	6.61	5.98	5.46	3.69	4.78	4.79	5.14
	Median		91.79	105.75	107.74	109.15	86.95	101.21	101.17	94.83
	Std. Deviation		20.32	23.82	21.55	19.69	13.30	17.22	17.28	18.55
	Variance		4.13	5.67	4.64	3.88	1.77	2.97	2.99	3.44
	Skewness		136.43	110.05	75.30	69.83	-23.63	-11.42	24.22	20.59
	Std. Error of		61.63	61.63	61.63	61.63	61.63	61.63	61.63	61.63
	Kurtosis		223.20	199.17	101.58	63.13	-25.02	-62.01	126.60	68.42
	Std. Error of		119.09	119.09	119.09	119.09	119.09	119.09	119.09	119.09
	Range		76.71	90.85	82.49	73.82	46.98	57.43	70.32	71.35
	Percentile	25	81.38	86.30	92.84	92.95	74.63	88.53	89.75	88.43
		50	91.79	105.75	107.74	109.15	86.95	101.21	101.17	94.83
		75	99.25	113.26	121.56	120.32	94.84	118.68	108.76	106.60
HR (bpm)	Mean		-1.88	-3.47	-3.35	-2.77	-1.05	-2.89	-2.09	-1.72
	Std. Error of Mean		0.62	0.84	0.80	0.77	0.86	1.01	1.13	1.16
	Median		-1.69	-3.12	-3.75	-2.64	-1.84	-4.32	-2.13	-1.61
	Std. Deviation		2.39	3.27	3.11	2.98	3.32	3.90	4.37	4.47
	Variance		5.72	10.71	9.67	8.86	11.05	15.23	19.09	20.02
	Skewness		-1.08	-0.37	-0.37	-0.56	1.17	0.30	0.37	0.43
	Std. Error of		0.58	0.58	0.58	0.58	0.58	0.58	0.58	0.58
	Kurtosis		1.05	-0.64	-0.30	-0.35	2.26	0.00	0.85	2.13
	Std. Error of		1.12	1.12	1.12	1.12	1.12	1.12	1.12	1.12
	Range		8.18	10.93	11.29	10.49	12.76	14.87	17.83	19.19
	Percentile	25	-2.62	-6.23	-5.22	-4.83	-3.63	-5.73	-5.49	-3.75
		50	-1.69	-3.12	-3.75	-2.64	-1.84	-4.32	-2.13	-1.61
		75	0.40	-1.22	-0.91	-0.18	0.85	0.58	-0.18	0.58
RSP (B/Min.)	Mean		1.78	1.47	1.39	1.64	2.68	2.47	2.30	2.06
	Std. Error of Mean		1.02	0.99	0.89	0.82	0.85	1.21	1.35	1.26
	Median		1.60	1.35	0.89	1.54	2.64	2.31	2.04	1.07
	Std. Deviation		3.96	3.83	3.45	3.16	3.31	4.67	5.24	4.90
	Variance		15.66	14.70	11.91	10.00	10.96	21.81	27.49	24.00
	Skewness		0.27	0.59	0.77	0.66	-0.29	0.87	0.77	0.04
	Std. Error of		0.58	0.58	0.58	0.58	0.58	0.58	0.58	0.58
	Kurtosis		0.46	0.72	0.94	1.27	-1.28	1.37	-0.17	0.87
	Std. Error of		1.12	1.12	1.12	1.12	1.12	1.12	1.12	1.12
	Range		15.13	15.15	13.71	12.93	10.05	18.45	16.79	19.59
	Percentile	25	-0.97	-0.65	-1.27	-1.34	0.00	-0.77	-1.77	0.13
		50	1.60	1.35	0.89	1.54	2.64	2.31	2.04	1.07
		75	5.05	2.92	3.02	3.36	5.59	5.45	3.89	5.22
SC (log(µS+1))	Mean		0.07	0.07	0.08	0.08	0.14	0.13	0.12	0.09
	Std. Error of Mean		0.01	0.02	0.02	0.02	0.04	0.04	0.04	0.03
	Median		0.06	0.05	0.05	0.06	0.12	0.14	0.08	0.05
	Std. Deviation		0.05	0.07	0.08	0.08	0.14	0.15	0.14	0.12
	Variance		0.00	0.01	0.01	0.01	0.02	0.02	0.02	0.02
	Skewness		0.41	0.23	0.47	0.73	0.79	0.55	0.40	0.20
	Std. Error of		0.62	0.62	0.62	0.62	0.62	0.62	0.62	0.62
	Kurtosis		-1.38	-1.64	-1.27	-0.38	-0.46	-0.85	-0.72	-0.45
	Std. Error of		1.19	1.19	1.19	1.19	1.19	1.19	1.19	1.19
	Range		0.13	0.20	0.23	0.24	0.41	0.45	0.46	0.43
	Percentile	25	0.02	0.01	0.01	0.01	0.02	0.02	0.02	0.01
		50	0.06	0.05	0.05	0.06	0.12	0.14	0.08	0.05
		75	0.12	0.15	0.15	0.14	0.23	0.24	0.22	0.18
