# Supplementary material for: The Power of an Infant's Smile: Maternal Physiological Responses to Infant Emotional Expressions
Source: PLoS One. 2015 Jun 11;10(6):e0129672. doi: 10.1371/journal.pone.0129672 (PMC4465828; doi:10.1371/journal.pone.0129672)
Supplement: S4 Table — (PDF) [file pone.0129672.s007.pdf]

S4 Table. Results of two-way ANOVA (related to Fig. 2).

		Type III Sum of Squares	df	Mean Square	F	Sig.	Partial Eta Squared
BVPa (%)	Intercept	1037160.98	1	1037160.98	802.55	0.00	0.97
	Condition	1857.37	1	1857.37	1.44	0.24	0.00
	Phase	3875.57	3	1291.86	21.35	***0.00	0.46
	Phase*Condition	137.34	3	45.78	0.76	0.52	0.02
	Error	4357.55	72	60.52			
HR (bpm)	Intercept	693.26	1	693.26	16.75	0.00	0.37
	Condition	25.80	1	25.80	0.62	0.44	0.01
	Phase	48.26	3[a]	24.09	5.49	*0.00	0.16
	Phase*Condition	1.92	3[a]	0.96	0.22	0.80	0.01
	Error	245.97	84[a]	4.38			
RSP (B/Min.)	Intercept	467.12	1	467.12	7.98	0.00	0.22
	Condition	19.67	1	19.67	0.34	0.57	0.01
	Phase	2.94	3	0.98	0.30	0.82	0.01
	Phase*Condition	1.52	3	0.51	0.16	0.93	0.01
	Error	272.50	84	3.24			
SC (μS)	Intercept	0.98	1	0.98	22.42	0.48	0.47
	Condition	0.05	1	0.05	1.19	*0.04	0.03
	Phase	0.01	3[a]	0.00	2.29	0.13	0.07
	Phase*Condition	0.01	3[a]	0.01	4.89	*0.01	0.16
	Error	0.06	72[a]	0.00			

Phase= {Cry, Experimental-1, Experimental-2, Experimental-3}, Condition={Neutral, Smile}

Sig.: Significance Probability, df: Degree of Freedom, $*p<0.05$, $***p<0.001$

a)The Greenhouse-Geisser adjustment was applied to correct for sphericity. Table shows uncorrected degrees of freedom.
