# Supplementary material for: The Power of an Infant's Smile: Maternal Physiological Responses to Infant Emotional Expressions
Source: PLoS One. 2015 Jun 11;10(6):e0129672. doi: 10.1371/journal.pone.0129672 (PMC4465828; doi:10.1371/journal.pone.0129672)
Supplement: S5 Table — (PDF) [file pone.0129672.s008.pdf]

S5 Table. Descriptive statistics of psychological measures at response points (pre-cry and post-experimental phases).

			Condition			
			Neutral (n=15)		Smile (n=15)	
	**Source**		Pre-Cry	Post-Experimental	Pre-Cry	Post-Experimental
PANAS						
Positive	Mean		2.19	2.03	2.49	2.33
	Std. Error of Mean		0.19	0.22	0.19	0.21
	Median		1.88	1.88	2.25	2.38
	Std. Deviation		0.75	0.87	0.75	0.81
	Variance		0.56	0.76	0.56	0.66
	Skewness		0.59	0.66	-0.07	0.40
	Std. Error of		0.58	0.58	0.58	0.58
	Kurtosis		-0.87	0.27	-0.96	-1.11
	Std. Error of		1.12	1.12	1.12	1.12
	Range		2.38	3.13	2.50	2.50
	Percentile	25	1.50	1.38	2.00	1.63
		50	1.88	1.88	2.25	2.38
		75	3.00	2.75	3.13	3.13
Negative	Mean		1.25	2.09	1.48	1.77
	Std. Error of Mean		0.11	0.22	0.15	0.26
	Median		1.13	2.13	1.25	1.13
	Std. Deviation		0.43	0.86	0.56	1.03
	Variance		0.18	0.74	0.32	1.05
	Skewness		2.39	0.56	1.13	1.09
	Std. Error of		0.58	0.58	0.58	0.58
	Kurtosis		5.36	-0.60	-0.06	-0.42
	Std. Error of		1.12	1.12	1.12	1.12
	Range		1.50	2.75	1.63	2.88
	Percentile	25	1.00	1.38	1.00	1.00
		50	1.13	2.13	1.25	1.13
		75	1.25	2.63	1.75	2.88
