# Supplementary material for: The Power of an Infant's Smile: Maternal Physiological Responses to Infant Emotional Expressions
Source: PLoS One. 2015 Jun 11;10(6):e0129672. doi: 10.1371/journal.pone.0129672 (PMC4465828; doi:10.1371/journal.pone.0129672)
Supplement: S6 Table — (PDF) [file pone.0129672.s009.pdf]

S6 Table. Results of two-way ANOVA for Table 3.

		Type III Sum of Squares	df	Mean Square	F	Sig.	Partial Eta Squared
PANAS							
Positive	Intercept	307.13	1	307.13	270.19	0.00	0.90
	Condition	1.35	1	1.35	1.19	0.29	0.00
	Response point	0.38	1	0.38	2.96	0.10	0.10
	Response point* Condition	0.00	1	0.00	0.00	1.00	0.00
	Error	3.56	28	0.13			
Negative	Intercept	162.94	1	162.94	210.60	0.00	0.88
	Condition	0.03	1	0.03	0.04	0.84	0.00
	Response point	4.75	1	4.75	12.81	*0.00	0.29
	Response point* Condition	1.17	1	1.17	3.16	0.09	0.07
	Error	10.37	28	0.37			

Response point= {Pre-Cry phase, Post-Experimental phase}, Condition={Neutral, Smile}

Sig.: Significance Probability, df: Degree of Freedom, *p<0.05
